# The Effectiveness and Exploratory Cost-Effectiveness of Regular Meditation for Improving Quality of Life: Protocol for a Prospective Longitudinal Cohort Study

**DOI:** 10.2196/85110

**Published:** 2026-07-17

**Authors:** Lillian Ofori Ward, Cate Bailey, Prai Wattanatakulchat, Benjamin Stone, Alexander Burger, Deanna Kaplan, Santiago Jose Arconada Alvarez, Cullan Joyce, Ana Eclair, Nicholas T Van Dam, Julieta Galante

**Affiliations:** 1 Contemplative Studies Centre, Melbourne School of Psychological Sciences, The University of Melbourne, Level 1, Melbourne Connect, 700 Swanston Street Victoria, Parkville, Victoria, 3010, Australia; 2 Melbourne Health Economics, School of Population and Global Health, The University of Melbourne, Melbourne, Victoria, Australia; 3 Department of Family and Preventive Medicine, Emory University School of Medicine, Emory University, Atlanta, GA, United States; 4 Department of Spiritual Health, Emory Healthcare, Emory University, Atlanta, GA, United States

**Keywords:** meditation, mindfulness, quality of life, well-being, adverse effects, cost effectiveness, spiritual, secular, mental health, health economics, costs, Buddhist

## Abstract

**Background:**

In recent decades, millions of people globally have taken up Buddhist spiritual and secularized meditation practices, such as mindfulness, with the aim of improving their quality of life and well-being. Practitioners are recommended to continue meditating regularly for the long term; however, the effects of regular meditation practice after introductory instruction remain scientifically underexplored.

**Objective:**

This protocol aims to investigate whether regular meditation practice improves quality of life and mental well-being and whether its effects vary between secular and spiritual practitioners. We will also explore the cost-effectiveness of meditation in terms of quality of life and the incidence and functional impact of any adverse effects associated with the practice.

**Methods:**

We will conduct a prospective longitudinal cohort study of 600 beginner meditators in Australia, New Zealand, the United States, and the United Kingdom over a 1-year period. Meditation practice frequency and duration will be reported weekly through the ambulatory assessment app Fabla (Emory University), with sampling of the primary outcome of quality of life and secondary outcome of mental well-being at monthly intervals. Practice characteristics, including secular versus spiritual practice, will also be reported monthly, as will the costs of meditation and mental health care and the incidence, severity, and duration of any adverse effects. Potential confounders, including baseline mental health symptoms, social support, and sociodemographics, will be controlled for in linear mixed models. An incremental cost-effectiveness ratio will be calculated, and sensitivity analyses will be conducted.

**Results:**

Data collection began on October 28, 2025, after this study was first submitted on October 1, 2025. As of May 14, 2026, 341 participants have completed baseline measures. We expect results to be published in January 2028.

**Conclusions:**

Results of this investigation will illuminate the impact of meditation as it is currently practiced in natural contexts, and inform clinicians about whether and how regular meditation may be an effective tool for improving quality of life.

## Introduction

### Background

In the past few decades, millions of people globally have taken up Buddhist-inspired forms of meditation practice, either as a spiritual practice within a traditional setting or as a secular practice within contemporary contexts [1]. Whether the context is spiritual or secular, supporting health and well-being is one of the main aims among those practicing meditation [1-3]. In Buddhist traditions, meditation is considered a lifetime pursuit that requires years of prolonged practice to accumulate tradition-specific insights [4] and thus achieve greater quality of life in a contemporary sense. Buddhist-inspired secularized meditation programs have become increasingly popular as a tool for promoting quality of life and well-being [5]. The most prominent examples of these are mindfulness-based programs (MBPs), which typically offer introductory courses lasting several weeks, including a near-daily home-based practice component, and which encourage practitioners to continue regular practice indefinitely to maintain and strengthen quality of life and well-being [6,7]. An estimated half of the practitioners follow the recommendation and continue their practice regularly beyond course completion [8,9]. However, the effects of regular meditation practice after introductory courses remain much less explored than the effects immediately following the intervention training [10]. With millions of practitioners globally, it is imperative to understand the effects of regular meditation practice for improving quality of life beyond the initial instruction and whether it is a safe and economically viable intervention over time.

### The Current Evidence

There is strong evidence supporting the immediate effects of secular MBPs on quality of life and mental well-being [11-14]. Several longitudinal studies have also assessed whether salubrious effects persist after course completion. Some suggest maintained benefits after as long as 4 years [9,15-17]; however, others have reported decreased benefits over one and 3 years [18,19]. While benefits of MBPs may be long-lasting, it is unclear whether they can be attributed to continued meditation practice, given that most longitudinal studies have not monitored whether the participants continued their practice beyond the instruction period. Findings from some clinical populations have indicated a decrease in the frequency [20] and duration [21] of home practice over follow-up periods of 1-12 months post-MBP.

Mixed preliminary evidence of a relationship between meditation practice and well-being has been found in randomized controlled trials (RCTs) and longitudinal studies that measured meditation practice following MBPs. Our RCT assessing the effects of an MBP on university students’ mental well-being found improvement postintervention [22] and up to a year later [23], compared with a passive control group. This trial tracked meditation practice after course completion and found that benefits increased with practice [23]. In another longitudinal study, students’ practice of mindfulness skills was positively associated with better mental well-being 3 months after the MBP [24]. On the other hand, a meta-regression failed to show evidence that longer or more intense MBPs predict better psychological outcomes [25]. A study with a 4-year follow-up also found continued practice in mindfulness meditation was not related to mental well-being [17]. However, continued practice was measured by a question at follow-up, rather than by tracking the amount of meditation over the intervening period. To appropriately assess the effect of meditation practice on long-term well-being outcomes, a longitudinal study that regularly and accurately assesses the amount of meditation practice is needed.

### Meditation in Spiritual and Secular Contexts

The evidence supporting meditation practice within spiritual contexts’ impact on well-being is much less developed than for secular interventions, probably because the latter are easier to incorporate into countries’ modern institutional settings. While spiritual and secular Buddhist meditation practices look similar, their contexts are often quite different, which may lead to different outcomes. Spiritual traditions usually integrate their philosophy or teachings into their practice, which may influence how practitioners engage with meditation. Spiritual traditions often recommend practitioners practice within moral guidelines like precepts as supports for practice and use exemplars (the Buddha or teachers) to orient practitioners [26,27]. Spiritual contexts also offer communities of practice, regular meditation classes, and dedicated practice spaces to support practitioners’ regular meditation after initial instruction; these are absent in most secular settings. Moreover, while secular programs, such as MBPs, are explicit about their aim to cultivate health and well-being, traditional forms of Buddhist meditation do not primarily aspire to produce health and well-being outcomes as their central goal [28]. Instead, they aim for spiritual liberation, insight into the nature of reality, and freedom from suffering (dukkha) in the Buddhist sense [29]. Increased quality of life and mental well-being are still to be expected as a side effect or fruit along the path; however, and are sought after by many contemporary spiritual practitioners [3].

While there is some evidence of well-being and mental health benefits from spiritual meditation practices [30], to our knowledge there is no longitudinal empirical evidence on the long-term effects of prolonged spiritual meditation practice. A cross-sectional survey by our team including both secular and spiritual meditation practitioners with varying lifetime practice history found a dose-response plateau between historical practice hours and psychological well-being outcomes after 500 hours of practice [31]. This survey also found that meditating at a regular time each day was associated with positive outcomes, supporting the hypothesis that regularity of practice may be more impactful than intense but irregular sets. Follow-up surveys at 2 months and 2-4 years with this same sample found both practice dose, that is, hours per month, and practice frequency significantly predicted improvements in life satisfaction, affect, and psychological distress [32]. However, these participants’ varying lifetime practice history introduces possible survivor bias effects (ie, only those who had already been benefiting from regular practice over the years signed up to complete these surveys) and obscures the effects of initiating a regular practice. Moreover, this study was not preregistered, included very heterogeneous types of meditation practice, and used a rough estimate of practice during the 2-4 year follow-up. To address these gaps, a preregistered longitudinal study focused on those initiating a regular practice is required.

To our knowledge, there is only one study that compares the outcomes of an explicitly spiritual meditation practice to a control group that includes secular meditation practices [33]. In this study of 31 participants, a program (Dharma in Daily Life) that emphasized both regular meditation and integration of Buddhist teachings showed greater improvement in subjective well-being (but not quality of life) than continued meditation practice as usual over a 2-year intervention period. However, while the meditation as usual group included secular practices, it also contained participants who engaged in some other spiritual meditation practices (eg, Vipassana and Zen), and all participants in both groups had some familiarity with Buddhism. A further study is necessary to disentangle the potential moderating effects of spiritual and secular meditation contexts on quality of life and mental well-being.

### Cost-Effectiveness of Meditation

If effective, regular meditation could form the basis of scalable and accessible public health interventions, as it requires relatively few economic resources [34]. Previous research shows some evidence that MBPs are generally cost-effective compared to usual care, specifically for quality of life and mental health [34-36], though there are some exceptions [37]. Moreover, it has been argued that the cost-effectiveness of MBPs may rely on practice adherence and may only be sustained with ongoing practice over time [35,36,38]. Since most studies have not taken into account whether the participants continue their practice after initial program completion or measured their ongoing meditation-related costs, it is yet to be established whether continued meditation practice is required to maintain cost-effectiveness [35,36]. To inform public and individual health decision-making, a longitudinal study that monitors continued meditation practice after initial instruction, as well as its associated costs and effects, is needed.

### Adverse Effects

There is growing recognition that for some practitioners, regular meditation practice can lead to harm [39-41]. Previous studies have documented a wide range of intense and unexpected experiences as a result of meditation practice, such as strong emotions, traumatic re-experiencing, and altered perceptions [42,43]. Such experiences can generate functional impairment and sometimes become clinically relevant [44]. Intense and unpleasant experiences are estimated to be encountered by 31%‐58% of meditation practitioners, depending on the measurement instrument, with about 9% of practitioners experiencing functional impairment [41]. There is some evidence indicating that adverse effects occur more often after initial instruction, in periods when secular practitioners typically have less or no support from teachers and peers [45]. Indeed, incidences of unpleasant and adverse effects are more commonly reported by practitioners with prolonged practice [41,42,44].

These findings have raised crucial questions about how frequently, and for whom, harm is experienced in meditation practice. Current evidence on the adverse effects of regular meditation practice comes from cross-sectional studies, which do not allow effects to be captured accurately due to memory and self-selection biases [44] or for their specific context or timing to be documented [46]. To address these concerns, a longitudinal study that assesses the incidence and impact of meditation-related adverse experiences as they arise during regular practice is needed.

### This Study

This study seeks to investigate the long-term effects of meditation practice on quality of life and well-being in current ecological contexts through longitudinal examination of a cohort of beginner meditators who intend to continue practicing over one year. Specifically, this study aims to estimate the effectiveness of regular meditation practice after initial instruction for promoting quality of life (Aim 1, primary outcome) and mental well-being (Aim 2). We hypothesize that the amount of regular meditation practice during approximately the first year after initial instruction will predict practitioners’ quality of life, such that more regular practice predicts greater quality of life (Hypothesis 1). We also hypothesize that the amount of regular meditation practice during approximately the first year after initial instruction will predict practitioners’ mental well-being, such that more regular meditation will predict greater mental well-being (Hypothesis 2).

The study further aims to test whether the meditation tradition being secular or spiritual moderates any effects on quality of life (Aim 3). We hypothesize that whether participants’ meditation practice is predominantly secular or includes engagement with spiritual practices will moderate any effects on quality of life (Hypothesis 3, nondirectional). We further aim to test whether the meditation practice being secular or spiritual moderates any effects on mental well-being (Aim 4). Mental well-being captures the flourishing spectrum of mental health, and there may be moderation effects detectable in this domain that would not be evident for quality of life [33], either due to a lack of effect or because those relationships are obscured by potential ceiling effects in negatively worded quality of life measures. We hypothesize that whether the meditation tradition is secular or spiritual will moderate any effects on mental well-being (Hypothesis 4, nondirectional).

The study also aims to explore the cost-effectiveness of regular meditation practice on quality of life (Aim 5). Given that this is a cohort study and we will not have a control group as we would in an RCT, we will conduct an exploratory economic analysis using a comparator group of those not continuing with meditation compared to those that are, accounting for confounding factors.

Finally, the study aims to document the incidence and impact on daily function of any challenging, difficult, or distressing meditation-related experiences (Aim 6). Meditation-related adverse effects (MRAEs) will be defined as any challenging, difficult, or distressing meditation-related experiences that impact everyday functioning. Our aims, hypotheses, and possible interpretations are summarized in Table 1 below.

**Table 1.** Protocol Design table.

Question	Hypothesis	Sampling plan (eg, power analysis)	Analysis plan	Interpretation given to different outcomes
Does regular meditation practice after initial instruction increase quality of life?	The amount of meditation practice during approximately the first year predicts practitioners’ quality of life, such that a greater amount leads to greater quality of life.	A total number of 522 gives 95% power to detect the variance in quality of life (EQ-HWB-9^a^) explained by monthly meditation practice observed in linear mixed model analysis, at a 2-tailed significance level of 0.05, accounting for an estimated 20% loss to follow-up.	Linear mixed model of the fixed effect of the amount of meditation in the past month on quality of life in the past week (EQ-HWB-9), controlling for the confounding factors of age, caring status, education, mental health diagnosis, commitment to meditation, and membership of a practice support community at baseline.	A positive/negative and statistically significant association between the amount of meditation in the past month on quality of life and in the past week will suggest that regular meditation practice after initial instruction increases/decreases quality of life in a linear fashion. A nonsignificant association will suggest an absence of evidence for either of these effects.
Does regular meditation practice after initial instruction increase mental well-being?	The amount of meditation practice during approximately the first year predicts practitioners’ mental well-being, such that a greater amount leads to greater mental well-being.	A number of 242 gives 95% power to detect the variance in mental well-being (SWEMWBS^b^) explained by monthly meditation practice observed in linear mixed model analysis, at a 2-tailed significance level of 0.05, accounting for an estimated 20% loss to follow-up.	Linear mixed model of the fixed effect of the amount of meditation in the past month on mental well-being in the past 2 weeks (SWEMWBS), controlling for the confounding factors of age, caring status, education, mental health diagnosis, commitment to meditation, and membership of a practice support community at baseline.	A positive/negative and statistically significant association between the amount of meditation in the past month on mental well-being in the past 2 weeks will suggest that regular meditation practice after initial instruction increases/decreases mental well-being in a linear fashion. A nonsignificant association will suggest an absence of evidence for this.
Is the effect of continued meditation practice after initial instruction on quality of life moderated by whether a practitioner’s meditation tradition is spiritual or secular?	Whether the meditation tradition is secular or spiritual will moderate any effects on quality of life (nondirectional)	Sampling plan follows hypothesis 1, with an additional 20%, n=627, powered to detect effect of f^2^=0.0318	Linear mixed model of the fixed effect of the amount of meditation in the past month and the interaction effect of tradition spirituality on quality of life in the past week (EQ-HWB-9), controlling for the confounding factors of age, caring status, education, mental health diagnosis, commitment to meditation, and membership of a practice support community at baseline.	A positive/negative statistically significant beta coefficient for this interaction will indicate that regular meditation practice within a spiritual tradition will have a larger/smaller effect on quality of life than practicing within a secular tradition. A nonsignificant association will suggest absence of evidence for this.
Is the effect of continued meditation practice after initial instruction on mental well-being moderated by whether a practitioner’s meditation tradition is spiritual or secular?	Whether the meditation tradition is secular or spiritual will moderate any effects on mental well-being (Hypothesis 4, nondirectional).	Sampling plan follows hypothesis 2, with an additional 20%, n=291, powered to detect effect of f^2^=0.045	Linear mixed model of the fixed effect of the amount of meditation in the past month and the interaction effect of tradition spirituality on mental well-being in the past 2 weeks (SWEMWBS), controlling for the confounding factors of age, caring status, education, mental health diagnosis, commitment to meditation, and membership of a practice support community at baseline.	A positive/negative statistically significant beta coefficient for this interaction will indicate that regular meditation practice within a spiritual tradition will have a larger/smaller effect on mental well-being than practicing within a secular tradition. A nonsignificant association will suggest absence of evidence for this.
What is the association between the costs and outcomes of meditation practice?	No hypothesis	Our sampling plan will follow hypothesis 1.	We will conduct an exploratory cost-effectiveness analysis to compare effects (quality of life) with a range of costs (costs of mental health support, costs associated with meditation practice)	Results will be presented as an ICER^c^ with associated sensitivity analysis, as outlined above.
What is the incidence and functional impact of meditation-related adverse effects	No hypothesis	—^d^	Proportion and CIs of the frequency, severity of impairment on daily function, and duration of impairment of initial meditation-related challenging experiences and adverse effects (MRAEs)	—

a EQ-HWB-9: 9-item EuroQoL Health and Wellbeing.

b SWEMWBS: Short Warwick-Edinburgh Mental Well-Being Scale.

c ICER: incremental cost-effectiveness ratio.

d Not applicable.

## Methods

### Overview

We will conduct a prospective longitudinal cohort study. Participants will be recruited online. We will apply best cohort study recruitment practices using meditation teachers and center networks [47]. After providing informed consent, participants will be requested to complete a series of self-report measures via online surveys hosted on the Qualtrics (Qualtrics International Inc) survey platform [48] and delivered through the ambulatory assessment mobile phone app Fabla (Emory University) [49], which will additionally support the collection of voice recordings within the app. This capability will be leveraged for participant verification, as described below, and for future qualitative analysis of the impact of meditation practice. There will be assessment time points at baseline and one year, and at weekly and monthly intervals in between, as shown in Table 2. For each survey, participants will receive mobile phone push notifications and email reminders. Example in-app images of the participant experience of data collection are displayed in Multimedia Appendix 1.

**Table 2.** Timeline from participant commencing the study.

Time	Start	Day 1	Day 2	Day 3	Day 4	Day 5‐11	Day 34	After 2 months of no practice reports	12 months
Action	Expression of interest survey	Consistent response check survey	Baseline survey	Fabla (Emory University) download survey	Fabla induction measures	Commence weekly measures	Commence monthly measures	Triggered follow-up survey	Outcome measurement
Access method	Advertising or website links	Email	Email	Email	Fabla	Fabla	Fabla	Email	Email

### Ethical Considerations

This study was approved by the Science, Technology, Engineering, Mathematics, and Medicine (STEMM 3)–Greater Than Low Risk (GTLR) human research ethics committee at the University of Melbourne on August 12, 2024 (reference: 2024-30046-56865-3). All participants will provide informed consent. Participants will be compensated at US $16 per hour rate for each baseline, monthly, and yearly survey completed and via entries in a gift card draw for each approximately 2-minute weekly survey.

### Participant Selection Criteria

We will recruit beginner meditators who have completed initial instruction in MBPs and 2 Buddhist traditions that have informed MBPs, Theravada, and Mahayana [50]. While all these traditions emphasize a focus on observing experiences and one’s responses to experiences, spiritual traditions often interpret these processes via specific lenses (eg, the Four Noble Truths in Theravada) and soteriologies that affect implicit and explicit motivations for a practice [51]. In addition, each tradition has exclusive introspective and reflective tools (eg, koans in Zen within Mahayana) that may be applied within meditation practices or in other aspects of life. Furthermore, the practice, social, and community contexts vary widely (eg, spaces, teachers, rituals, and other activities). The inclusion of these traditions will facilitate rich insights into the effects of diverse settings while keeping a meaningful focus.

Participants will be included if they are adults aged 18 years or older; reside in Australia, New Zealand, the United Kingdom, or the United States; have completed an initial introductory meditation course, initial instruction, or regular practice (at least 40 minutes per week) for at least a month in one of the eligible traditions (Buddhist Mahayana or Theravada or mindfulness meditation, including MBPs and apps that incorporate mindfulness meditation) in the last 4 months; and if they intend to continue practicing meditation regularly in the eligible meditation tradition. We are recruiting participants with some meditation experience rather than those with no lifetime practice hours to examine a naturalistic sample of people who independently seek out and continue meditation practice. This sample will also support the study retaining participants who continue practicing meditation for one year, and therefore have sufficient power in our primary analysis, as significant dropout is common in the first weeks of meditation practice [52-55].

Participants will be excluded if they have less than one month of experience in their meditation practice (with the option to defer enrollment until they satisfy this criteria), if they have regular meditation experience (meditating at least 40 minutes per week) for one month or more in any tradition prior to the last 4 months, or if they are monastics or have a similar formal role in a religious community and/or they reside in a religiously affiliated dwelling (eg, monastery).

### Participant Verification

The study uses a combination of methods to both discourage nongenuine participants and to verify that those participants who are recruited are genuine. Entry into the study involves 4 Qualtrics surveys delivered on consecutive days, followed by download of the app Fabla, which is restricted to Australia, New Zealand, the United Kingdom, or the United States, before commencing the weekly survey schedule, as shown in Table 3. The first expression of interest survey will exclude participants who fail Qualtrics internal bot checks and who are detected to be outside our recruitment countries. The second survey will repeat date of birth questions from the expression of interest to detect inconsistent responses.

**Table 3.** Schedule of measures.

Measures	Baseline	Weekly	Monthly	1 year
Outcome measures				
Health and well-being (EQ-HWB-S^a^)	✓		✓	✓
Mental well-being (SWEMWBS^b^)	✓		✓	✓
Independent variables				
Meditation practice frequency and duration		✓		
Meditation practice characteristics	✓		✓	✓
Secular or spiritual practice tradition	✓		✓	✓
Covariates				
Meditation history	✓			
Sociodemographics	✓			
Physical and mental health status	✓			✓
Other variables				
Cost of meditation practice	✓		✓	✓
Cost of mental health care use	✓		✓	✓
Meditation-related adverse effects scale (MRAES)	✓		✓	✓

a EQ-HWB-S: EQ Health and Wellbeing—Short version.

b SWEMWBS: Short Warwick-Edinburgh Mental Well-Being Scale.

After we have received 7 separate survey responses from participants, their IP and internet service provider (ISP) will be inspected. This IP inspection identifies participants whose IP addresses change on each survey, which indicates possible “residential proxy” use, those who have IP addresses associated with a known virtual private network (VPN), those who have more than 2 ISPs associated with their IPs, and those who have IP addresses associated with a data center (eg, Amazon Web Services).

Participants who are identified by these IP inspections will be manually screened by listening to voice recordings they will provide in Fabla. Those participants who we are not able to verify through this process will be contacted and offered an online interview. Prospective participants who do not respond after follow-up, refuse an interview or alternative contact method, or are suspected to be nongenuine during an interview will be considered nongenuine, excluded from further participation, and have their data removed.

### Measures

A schedule of measures is shown in Table 3 and described in detail below.

Meditation practice frequency and duration will be assessed weekly. Participants will be asked how many days they practiced in the past week, the average number of sessions per day, and the average minutes per session. This is a weekly modification of the timeline followback method, which is the gold standard for reporting in substance use literature, and has been validated for exercise reporting [32] and online administration [56]. We have also validated this question format against a log of meditation timer practice data [32]. Weekly measurement supports accurate reporting in similar methods [57], while allowing us to calculate the amount of meditation each month. This will be our independent variable.

Meditation practice characteristics, including type (eg, meditation with movement such as walking meditation or mindful observation such as observing thoughts or emotions), guided versus unguided sessions (eg, “I work with a teacher or instructor” or “My practice is self-guided”), and social context of their practice (eg, “I practice in a group” or “I practice alone”), will be recorded monthly. These measures will be used to describe the sample by reporting the frequency of each practice type and context reported by participants over one year.

Secular or spiritual meditation practice will be assessed at baseline and monthly by a single item (“In the past month, what meditation traditions have you practiced in?”). Participants will be divided into 2 groups at follow-up; those who both nominated secular meditation as their tradition at baseline and reported a maximum of one month of practice in any spiritual tradition over the follow-up period, versus all participants who practiced in spiritual traditions for 2 months or more during the year. This division will facilitate investigating if those meditation practitioners who practice in spiritual traditions experience the same effects as secular practitioners, while allowing for some dabbling in different traditions by participants. This will be a covariate.

Commitment to meditation practice will be assessed at baseline with a single item (“How committed are you to practicing meditation?” Rate 1‐10). This will be a covariate.

Community of practice membership will be assessed by a single item and recorded at baseline and one year (“Are you an active member of a community of practice [eg, sangha, meditation group, or online communities]? Yes/No”). This will be a covariate.

Age in years will be recorded at baseline. This will be a covariate.

Caring status, including type of caring relationship, will be assessed at baseline. This will be a covariate.

Education will be assessed by a question asking, “What is the highest level of education you have completed?” and will be recorded at baseline. This will be a covariate.

Mental health diagnosis will be assessed by a single item at baseline (“Have you ever been diagnosed with a mental illness by a clinician?” Yes/No/Prefer not to say). This will be a covariate.

The cost of meditation practice will be assessed through self-reported questions asking participants whether they have spent any money related to their meditation practice, including on classes, courses, retreats, smartphone apps, resources such as mats, books, and clothes, and donations to meditation teachers or centers. These questions will be asked at baseline, monthly, and one year later.

The cost of mental health care use will be assessed via questions on type, frequency, and costs of psychological support. Specifically, participants will be asked, “In the past month, how many times did you see a… psychologist, psychiatrist, social worker, counselor, or other.” Participants will then be asked how they paid for this mental health service use (eg, “Out-of-pocket” and “Publicly funded”) and their out-of-pocket expenditure on mental health services in the past month. These questions will be asked at baseline, monthly, and one year. This will be used in the economic evaluation.

Quality of life will be assessed at baseline, monthly, and one year, using the 9-item EQ Health and Wellbeing (EQ-HWB-9) instrument [58]. The EQ-HWB-9 captures mental (eg, depression and cognition), physical (eg, pain), and social (eg, loneliness) aspects of quality of life for the past 7 days, on a 5-point Likert scale from 1 (No difficulty) to 5 (Unable). Previous responsiveness to change analysis indicates that the EQ-HWB-9 detects meaningful changes in both mental and physical health measures [59]. This will be our primary outcome. The EQ-HWB-9 also allows for the calculation of quality-adjusted life years (QALYs), a standard measurement in health decision-making, and thus allows for the comparison of both effectiveness and cost-effectiveness with many other interventions and standard treatments [60].

Mental well-being will be assessed at baseline, monthly, and one year using the Short Warwick-Edinburgh Mental Well-Being Scale (SWEMWBS) [61,62]. The SWEMWBS includes 7 items that assess mental well-being over the past 2 weeks, on a 5-point Likert scale from 1 (None of the time) to 5 (All of the time). This will be our secondary outcome. The items in the SWEMWBS are positively worded (eg, “I’ve been feeling optimistic about the future”) and aim to capture variation in the flourishing side of the well-being spectrum, which will supplement detailed variation in the quality of life deficits captured by the EQ-HWB-9. Previous analyses of responsiveness to change indicate that the SWEMWBS detects individually meaningful improvement across a variety of mental health measures [63].

MRAEs will be measured at baseline, one year, and monthly, using the 3-item MRAEs measures [42]. The first item asks participants to indicate the frequency of any challenging, difficult, or distressing meditation-related experiences, with options ranging from “never” to “frequently.” The second item assesses the level of impairment caused by these experiences on the participant’s ability to function, with options ranging from “not at all” to “severely.” The third item asks about the duration of the impairment, with options ranging from “1 day or less” to “1 year or longer.”

### Sampling Plan

#### Hypothesis 1

Sample size calculation for quality of life measured by the EQ-HWB-9 using repeated measures linear mixed modeling assumes three groups will be assessed with (1) low, (2) medium, and (3) high regular meditation dosage. Given the limited research on the effects of long-term regular meditation practice, calculating power is challenging. We have therefore used conservative power calculations that may err on the side of underestimating the power of our primary analysis but allow for use of the effect information and analytic tools that are available. We have conducted power analysis using 3 levels of variation in the predictor of monthly meditation amount, rather than the continuous variable we plan to use in our final analysis. This is to allow for the use of an analytic power calculation method for our repeated measures design, rather than simulation measures, which would be highly speculative with the limited real and independent data available for our outcome measure [64]. Ten measurement time points were also used, rather than the 12 that will be included in our analysis, as this is the maximum for GLIMMPSE (University of Florida and University of Colorado Denver) power analysis software [65].

Sample size calculation for quality of life measured by the EQ-HWB-9 using repeated measures linear mixed modeling assumes three groups will be assessed with (1) low, (2) medium, and (3) high regular meditation dosage. No increase in EQ-HWB-9 scores was predicted for the low meditation group [58]. For the high meditation group, we estimate a stepwise increase to a minimum of a 2.25-point EQ-HWB-9 increase (out of a possible 9‐45 points) from baseline to time point 5, corresponding to the change in EQ-HWB-9 from baseline to 6 months associated with improvement in mental health in a validation of the EQ-HWB-9 sensitivity to change [59]. We then predict a further 2.25-point increase to time point 10, for a total increase of 4.5 points. For the moderate meditation group, stepwise improvement was predicted with a minimum of a 2.25-point EQ-HWB-9 increase from baseline to time point 10. Baseline EQ-HWB-9 is set at 19.6, with an SD of 7.0, as per our nationally representative surveys [66]. Using GLIMMPSE [65] with 95% power and 0.05 2-tailed type 1 error rate, sample size was calculated at 435 participants. We estimated a 20% loss to follow-up over one year, bringing the sample size required to 522 participants.

#### Hypothesis 2

There is limited research available on the effects of long-term meditation on mental well-being using the 7-item SWEMWBS. However, there is published data available for the 14-item version (Warwick-Edinburgh Mental Well-being Scale; WEMWBS) for postinitial meditation instruction [22] and at one year follow-up [23]. These 2 mental well-being scales are highly correlated (0.954) [61], so we have used the data available for the WEMWBS in our power calculation.

Sample size analysis for the effect of meditation practice amount on mental well-being (WEMWBS) using repeated measures linear mixed modeling assumes three groups will be assessed with (1) low, (2) medium, and (3) high meditation dosages, with 10 measurement time points, as required for the use of GLIMMPSE [65] as an analytic power calculation method outlined above. The baseline mean WEMWBS is set at 49.61, the postintervention level found for the introductory meditation arm, and the SD of 8.88 [22] for all groups and time points. No increase in WEMWBS scores [62] is predicted for the low meditation group. For the moderate meditation group, we predict stepwise improvement of 3 points to a final time point mean of 51.06 (SD 8.88), corresponding to a one-year follow-up of an MBP, regardless of regular meditation rates after course completion [23]. For the high meditation group, we estimate stepwise improvement to an 8-point increase from baseline to final time point (mean 57.61, SD 8.88), representing a minimum individual detectable change that has been reported for the WEMWBS [63]. Using GLIMMPSE [65] with 95% power and 0.05 2-tailed type 1 error rate, the required sample size to detect these effects was 201 participants, 242 assuming a 20% loss to follow-up.

#### Hypotheses 3 and 4

There is limited research investigating whether meditation practice being spiritual or secular moderates the effect of practice time. Previous studies have shown evidence that engaging in regular spiritual mind-body practices may moderate the effect of an initial meditation program at 6 months, such that stress and depressive symptoms are only improved compared to treatment as usual for those individuals who do not regularly engage in spiritual mind-body practices [67]. However, this was not compared to similar secular meditation practices. Our prospective longitudinal study found that those with a spiritual goal for meditation practice obtained less benefit from their meditation practice for life satisfaction and positive affect [32]. Nevertheless, to our knowledge, the moderating effect of practicing within a spiritual tradition on quality of life or well-being as outcomes is yet to be investigated or observed over a one-year follow-up. Therefore, potential effect sizes for these analyses are highly uncertain. As such, to account for testing these hypotheses, we plan to increase our sample by an additional 20% from what is required for power to test our primary outcome (Hypothesis 1) to 627 participants. With this sample and our planned model, setting power to 0.95 and significance to 0.05 2-tailed, we will be powered to detect a moderation effect of f^2^=0.0352 [68].

### Analysis Plan

The statistical analyses used to test each of our hypotheses are described below and summarized in Table 2. We will conduct the exploratory cost-effectiveness analysis in Stata (StataCorp LLC; [69]) and all other statistical analysis in R (R Foundation for Statistical Computing; [70]). We will report descriptive statistics (mean and SD if normally distributed, median and range if not) of all the variables included in the analyses. For all the analyses involving linear regression, we will check if the assumptions of linear regression are met by inspecting the residual plots for linearity, homogeneity of variance, normality, independence of residuals, and collinearity. If basic assumptions are not met, we will attempt to meet them via variable transformation, and failing that, we will use robust regression methods.

To ensure we only analyze responses from genuine participants, we will exclude participants from the study who give impossible or mutually incompatible responses as soon as they are detected [71]. As the primary strategy to handle missing data, the longitudinal constrained analysis assumes that the probability of missing outcome data is not related to the missing data but to some of the observed data in the model (missing at random [MAR]). Our models will use a restricted maximum likelihood (REML) estimator to handle missing data. The use of the REML estimator will enable us to effectively handle the potential bias introduced by differential attrition, as it allows for the estimation of unbiased fixed-effects coefficients and appropriately accounts for the variability introduced by random effects.

In addition, we will perform sensitivity analysis to assess how robust our analysis of the primary outcome is to deviations from MAR. We will calculate censoring weights by investigating the baseline and time-varying predictors of attrition, including engagement and response to follow-up factors. We will then recalculate the primary analysis with inverse probability of censoring weighting [72].

For hypothesis 1, we will perform linear mixed model analysis of the fixed effect of the amount of meditation in the past month on quality of life in the past week (EQ-HWB-9), controlling for the potentially confounding baseline factors of age, caring status, education, mental health diagnosis, commitment to meditation practice, and membership of a meditation practice community at baseline. This model will allow us to assess how meditation as practiced by our participants under naturalistic conditions contributes to their quality of life, controlling for baseline differences related to time available for practice, symptom severity, motivation, and social support. If we detect an effect of meditation practice, we will perform sensitivity analysis to examine how robust this effect is to time-varying confounders by using inverse probability weighting of meditation in the last month to examine if the association would still be present if practice that month had varied independently of prior practice and benefit.

For hypothesis 2, we will perform linear mixed model analysis of the fixed effect of the amount of meditation in the past month on mental well-being in the past 2 weeks (SWEMWBS), controlling for the potentially confounding factors of age, caring status, education, mental health diagnosis, commitment to meditation practice, and active membership of a meditation practice community at baseline. If we detect an effect of meditation practice, we will again perform time-varying confounding sensitivity analysis with inverse probability weighting.

For hypothesis 3, we will add the variable capturing whether participants practiced secular meditation traditions (with no more than one month of reported practice in a spiritual tradition) versus engaging in spiritual traditions and its interaction term with the amount of meditation to the model for hypothesis 1. This initial analysis will allow us to assess whether predominantly secular meditation has a differential effect from meditation that incorporates spiritual practices, as practiced in real-life contexts, while retaining sufficient power. If we detect an effect, we will perform further exploratory analysis into what may be driving this difference. We will also describe the groups in terms of meditation practice characteristics, including proportion of practice types and social context.

For hypothesis 4, we will add the secular versus spiritual variable and its interaction term with the amount of meditation to the model for hypothesis 2.

To address aim 5, we will conduct an exploratory cost-effectiveness analysis using participants who did not continue meditating as a comparator to participants with ongoing meditation practice, while accounting for relevant confounding factors. We will use the EQ-HWB-9 to measure outcomes, applying index values from the United Kingdom pilot value set over time to create QALYs [60]. Costs will include those for mental health service use (psychological support) across one year plus costs of meditation practice. To calculate the incremental cost-effectiveness ratio (ICER) from a public health perspective, we will calculate the difference in costs between the low- and high-meditation dosage groups and divide by differences in QALYs. The ICER represents the additional cost of the intervention for each additional unit of health benefit gained. We will investigate the cost-effectiveness of the ICER against thresholds of the 4 included countries, as available, to determine how likely the intervention will be cost-effective from a public health perspective. Issues of confounding will be addressed through statistical adjustments as per the main analysis, and we will conduct relevant scenario analyses. We will conduct deterministic (one-way) sensitivity analysis to estimate the impact of uncertainty around effects by varying output and cost parameters, displayed on tornado graphs. We will conduct probabilistic sensitivity analysis to explore the combined parameter uncertainty over 10,000 iterations, displayed on a cost-effectiveness plane.

To address aim 6, we will estimate the incidence of participants first reporting adverse effects by reporting the proportion and confidence intervals of first adverse experiences that also impaired participants’ daily function. We will also report descriptive statistics of the frequency, severity of impairment of daily function, and duration of impairment of meditation-related challenging experiences and MRAEs.

## Results

The protocol for this study was preregistered on May 9, 2024 [73] (embargoed). This manuscript was originally submitted on October 1, 2025. We soft-launched to test our systems on October 28, 2025, and initiated substantial recruitment efforts in January 2026. As of May 14, 2026, 341 participants have completed baseline measures. We expect to complete data collection and publish results in January 2028.

## Discussion

This study examines the unfolding effects of continued meditation as currently practiced in ecological contexts. The longitudinal design allows us to investigate these effects in those who commence and sustain regular meditation practice. Given preliminary evidence that long-term meditation can lead to improved well-being [22], we hypothesize that the amount of regular meditation practice during approximately the first year after initial instruction will predict practitioners’ quality of life, such that more regular practice predicts greater quality of life. We also hypothesize that the amount of regular meditation practice during approximately the first year after initial instruction will predict practitioners’ mental well-being, such that more regular meditation predicts greater mental well-being. We anticipate these effects may be modest and partially explained by the impact of regularly practicing in a community, as group practice has been indicated to improve meditation’s impact on health goals [74,75]. If our study finds evidence for long-term meditation improving health and well-being, it will be in contrast to the limited evidence for its positive impact when participants are assigned or randomized to meditation instruction [38]. Together with previous research by our team [31,32], this may suggest that people’s choice to engage in meditation practice is an important factor for any benefit they may derive from it.

We also hypothesize that whether participants’ meditation tradition is secular or spiritual will moderate any effects on quality of life and mental well-being. It is difficult to anticipate a direction of effect given the scarce evidence base. If we detect a more positive effect of spiritual practices, we expect that this effect will be at least partially explained by social support, since spiritual meditation is more often carried out within well-established spiritual communities. Secular meditation practices have a relatively recent history compared to spiritual practices and have different objectives, while often lacking community practice elements. Secular meditation practices are growing quickly in application; however, it is important to understand the impact of disengaging meditation practices from spiritual contexts. For example, continued access to social support may be an important element in the benefit of spiritual meditation practices that should be considered in secular contexts.

The study also aims to conduct an economic evaluation in the form of an exploratory cost-effectiveness analysis to investigate costs and outcome comparisons associated with regular meditation practice. As this study is a cohort study and not an RCT where we would usually see cost-effectiveness analysis conducted, and as there is no control group, we plan to construct an exploratory cost-effectiveness analysis using a comparator group of those not continuing to meditate. This is a major limitation to the cost-effectiveness analysis proposed in this study.

There are further limitations regarding effects and costs. Although the EQ-HWB-9 has been shown to be valid for measuring quality of life in people who meditate [76], there are currently no country-specific value sets for the EQ-HWB-9 for Australia, New Zealand, or the United States. We will have detailed information on costs due to psychological care; however, we will need to estimate costs from this information for the relevant country’s health systems where costs are not out-of-pocket. This analysis is further exploratory on the basis of the direction of the effect. For instance, if, through partaking in meditation practice, participants decide to pursue mental health support, this will increase costs but may also increase outcomes. Hence, lowering mental health support costs may not be the objective here. We acknowledge the potential opportunity cost of meditation practice, and while we will be able to provide information on the time commitment needed to achieve benefit in our primary analysis, we are unable to address this in the current exploratory cost-effectiveness analysis. As the outcomes look at change in QALYs over one year, we will also not be able to take into account changes in effects and costs during this period for the base analysis. We may be able to do further exploratory analysis investigating the associations between costs and effects over time in a future study.

Finally, the study aims to estimate the incidence of MRAEs. We anticipate the incidence of MRAEs will be around 10%, in line with previous research [42]. We also expect the average impact on daily function to be small, on average, but that some participants will experience significant impairment to daily function. We plan to conduct a future mixed methods examination of MRAEs to explore their predictors and phenomenology.

This study is limited to those who are motivated to continue an initial meditation practice and to contribute to a yearlong research study. While this does allow us insight into meditation as it is currently practiced, these findings are limited to those who independently choose to meditate. As such, the study also does not control for the effects of expectancy or placebo, which may contribute to the benefit our participants derive from their meditation practice. Restricting inclusion to only participants familiar with meditation and intending to continue may somewhat mitigate variance from these effects.

## Supplementary material

10.2196/85110 Multimedia Appendix 1 In-app images from Fabla, showing participant experience of data collection.

## Abbreviations

EQ-HWB-9: 9-item EuroQoL Health and Wellbeing
GTLR: Greater Than Low Risk
ICER: incremental cost-effectiveness ratio
ISP: internet service provider
MAR: missing at random
MBP: mindfulness-based program
MRAE: meditation-related adverse effect
QALY: quality-adjusted life year
RCT: randomized controlled trial
REML: restricted maximum likelihood
STEMM: Science, Technology, Engineering, Mathematics, and Medicine
SWEMWBS: Short Warwick-Edinburgh Mental Well-Being Scale
VPN: virtual private network
WEMWBS: Warwick-Edinburgh Mental Well-Being Scale
