# Supplementary material for: The Effectiveness and Exploratory Cost-Effectiveness of Regular Meditation for Improving Quality of Life: Protocol for a Prospective Longitudinal Cohort Study
Source: JMIR Res Protoc. 2026 Jul 17;15:e85110. doi: 10.2196/85110 (PMC13378107; doi:10.2196/85110)
Supplement: Multimedia Appendix 1 [file resprot-v15-e85110-s001.pdf]

9:33

Weekly Goal

0/1

Weekly Survey: 0/1

Today's Entries

(Optional) Meditation Timer

Available until: 11:45 PM

Multiple submissions allowed

Meditation Diary

Available until: Jun 5, 2026

Study History Settings

9:34

In the past week, how many days of the week did you meditate (from 0 to 7)?

0 days

1 day

2 days

3 days

Finish

9:34

In the past week, how many sessions did you practise per day on average?

1

→

Powered by Qualtrics

Finish

9:34

In the past week, how many minutes per session did you practise on average?

30

→

Powered by Qualtrics

Finish

9:34

Question 2/2

Talk about your meditation this week, if you didn't meditate, talk about other experiences this week

Please try to talk without stopping for about 5 minutes as if you were sharing about your day with a friend. Do not worry about pauses, stutters, or having the right things to say.

Record My Response

Next

9:47

Thanks for your response!

Your input is incredibly valuable for our study's progress. We can't wait to hear from you again soon!

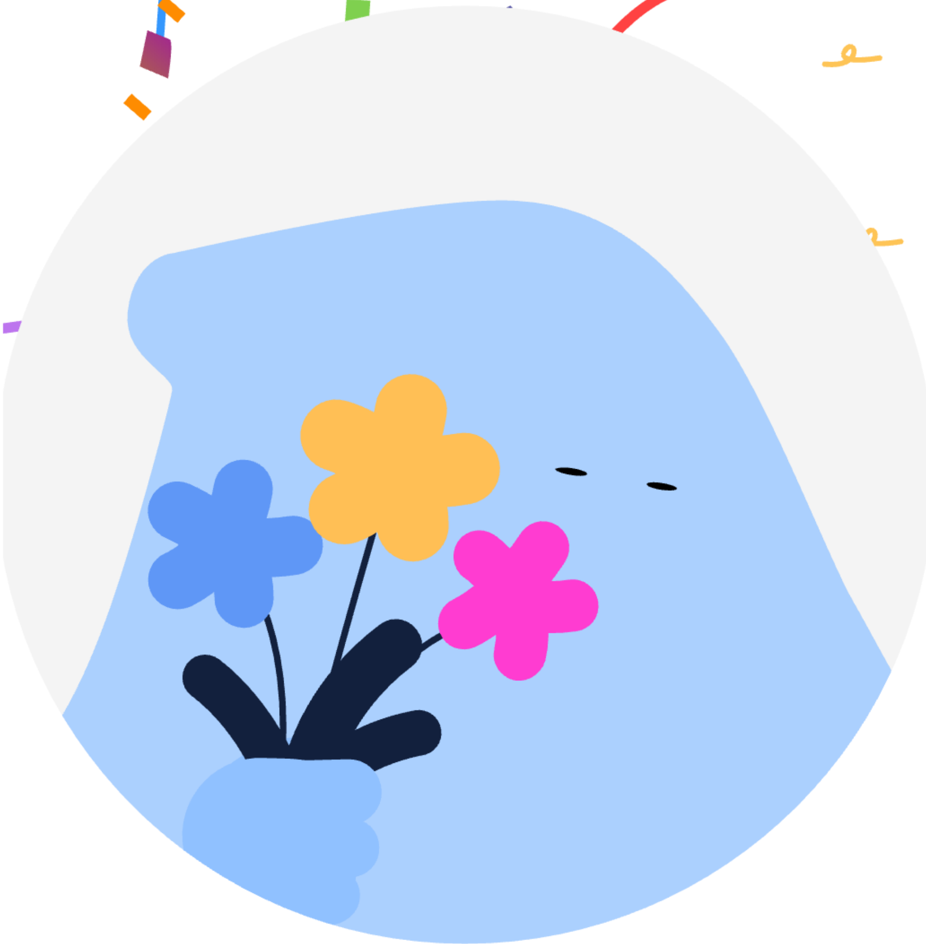

Return Home

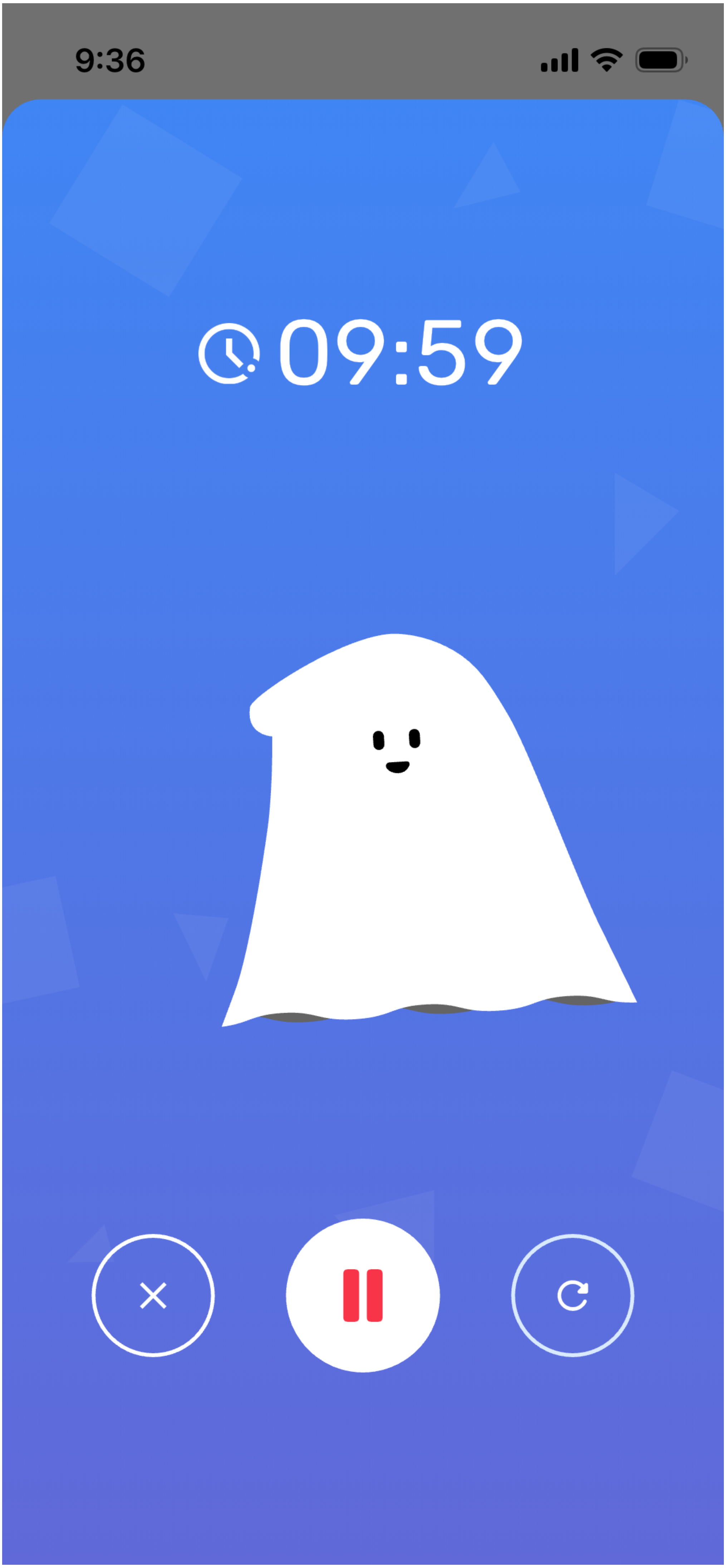
9:36
09:59

9:47

Weekly Goal

1/1

Today's Goal

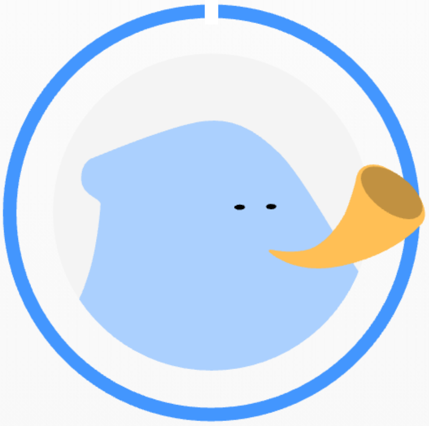

Weekly Survey: 1/1

Today's Entries

(Optional) Meditation Timer

✓ Submitted

Multiple submissions allowed

Study History Settings

9:47

Study Calendar

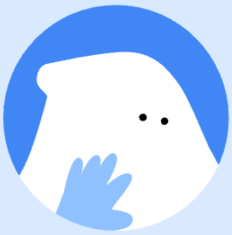

14 days active - You're doing GREAT!
Keep working towards the goals

Study Calendar

June 2026

M	T	W	T	F	S	S
1	2	3	4	5	6	7
8	9	10	11	12	13	14
15	16	17	18	19	20	21
22	23	24	25	26	27	28
29	30	1	2	3	4	5

9:47

Incentives Earned

$10.00

Compensation Details

Total Compensation

$115.00

Monthly Assessment #1

$0.00/$0.00

Monthly Assessment #2

$5.00/$5.00

Monthly Assessment #3

$0.00/$5.00
